# Fighting Celiac Disease: Improvement of pH Stability of Cathepsin L In Vitro by Computational Design

**DOI:** 10.3390/ijms241512369

**Published:** 2023-08-02

**Authors:** Anton O. Chugunov, Elena A. Dvoryakova, Maria A. Dyuzheva, Tatyana R. Simonyan, Valeria F. Tereshchenkova, Irina Yu. Filippova, Roman G. Efremov, Elena N. Elpidina

**Affiliations:** 1 M.M. Shemyakin and Yu. A. Ovchinnikov Institute of Bioorganic Chemistry, Russian Academy of Sciences, 117997 Moscow, Russia; duzheva.maria@mail.ru (M.A.D.); r-efremov@yandex.ru (R.G.E.); 2 L.D. Landau School of Physics, Moscow Institute of Physics and Technology (State University), 141701 Dolgoprudny, Russia; 3 A.N. Belozersky Institute of Physico-Chemical Biology, M.V. Lomonosov Moscow State University, 119991 Moscow, Russia; greenfire06@gmail.com (E.A.D.); elp@belozersky.msu.ru (E.N.E.); 4 Higher Chemical College of the Russian Academy of Sciences, D. Mendeleev University of Chemical Technology, 125047 Moscow, Russia; 5 Department of Chemistry, M.V. Lomonosov Moscow State University, 119991 Moscow, Russia; simoniantania@yandex.ru (T.R.S.); v.tereshchenkova@gmail.com (V.F.T.); irfilipp@belozersky.msu.ru (I.Y.F.); 6 Department of Applied Mathematics, National Research University Higher School of Economics, 101000 Moscow, Russia

**Keywords:** celiac disease, gluten intolerance, glutenase, acid stability, post-glutamine cleaving peptidase, cathepsin L, molecular dynamics, *Tribolium castaneum*

## Abstract

Roughly 1% of the global population is susceptible to celiac disease (CD)—inheritable autoimmune inflammation of the small intestine caused by intolerance to gliadin proteins present in wheat, rye, and barley grains, and called gluten in wheat. Classical treatment is a life-long gluten-free diet, which is constraining and costly. An alternative approach is based upon the development and oral reception of effective peptidases that degrade in the stomach immunogenic proline- and glutamine-rich gliadin peptides, which are the cause of the severe reaction in the intestine. In previous research, we have established that the major digestive peptidase of an insect *Tribolium castaneum*—cathepsin L—hydrolyzes immunogenic prolamins after Gln residues but is unstable in the extremely acidic environment (pH 2–4) of the human stomach and cannot be used as a digestive aid. In this work, using molecular dynamics simulations, we discover the probable cause of the pH instability of cathepsin L—loss of the catalytically competent rotameric state of one of the active site residues, His 275. To “fix” the correct orientation of this residue, we designed a V277A mutant variant, which extends the range of stability of the peptidase in the acidic environment while retaining most of its activity. We suggest this protein as a lead glutenase for the development of oral medical preparation that fights CD and gluten intolerance in susceptible people.

## 1. Introduction

Celiac disease (CD) is a hereditary autoimmune disorder characterized by chronic inflammation of the small intestinal mucosa accompanied by malabsorption due to persistent intolerance to the proline/glutamine(PQ)-rich plant storage proteins (prolamins) of wheat (gluten), rye and barley seeds [1]. This disease is not associated with age, can occur at any time and is diagnosed in 1–2% of the world’s population. Over time, there is a high risk of developing oncological and autoimmune diseases and infertility, as well as nervous disorders. There is currently no treatment for CD, and the only effective way for sufferers to stay healthy is to follow a strict gluten-free diet, which is difficult to adhere to due to the serious restrictions it imposes, leading to asocialization and depressive states. Patients on this diet are deficient in vitamins and minerals and are prone to anemia and osteoporosis. In addition, 0.5–13% of the world’s population is diagnosed with gluten intolerance without celiac disease (non-celiac gluten sensitivity, or NCGS) [2]. After eating foods with gluten, NCGS patients face the same problems as those with CD [3]. In this regard, various therapeutic strategies are being developed to combat gluten intolerance.

One particularly significant approach is enzyme therapy, that is, the intake with food of peptidase preparations that can cleave hardly hydrolyzable prolamin peptides. This approach eliminates the cause of the disease—undercleaved PQ-rich peptides (PQ content in prolamins represents ~65–80% of the total number of amino acids). Currently, a significant number of glutenases of various origins (bacteria, fungi, plants, insects) and families (serine, cysteine, metallopeptidases) capable of hydrolyzing toxic immunogenic prolamin peptides have been identified [4,5]. It has been found that proline-specific glutenases are not as effective as post-glutamine cleaving peptidases, and a combination of two different activities is much more attractive. However, at present, the range of such proposed preparations is very limited and consists of enzymes from different sources, including pathogenic bacteria, that are not adapted to each other, which limits the possibility of their use.

It is not only safety issues that hinder the promotion of enzyme preparations in the pharmaceutical market. A serious disadvantage of glutenases as possible therapeutic agents is their rapid inactivation and instability in the conditions of the human stomach, i.e., at low pH [4]. Attempts to stabilize enzymes through pharmaceutical modification, such as PEGylations and microencapsulation, have so far been very limited [6]. There are two principal strategies to obtain pH-stable glutenase: (1) take another pH-stable peptidase and switch its specificity to prolamins, or (2) take an effective glutenase and make it pH stable.

The first strategy is exemplified by the Kumamax oral protein therapeutic [7,8], which has recently passed through phase 1 clinical trials [9]. In this contribution, a protein design approach mediated by the Rosetta software suite was used to redesign the active site of the kumamolisin-As, known to be active at low pH. The engineered enzyme exhibited 116-fold greater proteolytic activity for a model gluten tetrapeptide than the native template enzyme, as well as an over 800-fold switch in substrate specificity toward immunogenic portions of gluten peptides.

In this work, we implement the second strategy, based on conferring pH stability to an enzyme that already has sufficient glutenase activity. Earlier, we have shown that the main digestive cathepsin L from the insect pest *Tribolium castaneum* (TcCathL1, Uniprot D6X519, NCBI NP_001164001) has high post-glutamine cleaving activity and is able to degrade 33-, 26-, 10- and 8-mer immunogenic prolamin peptides and their fluorogenic analogs [10,11]. Successful experiments were carried out both with the native enzyme isolated from the *T. castaneum* midgut extract [10] and with the recombinant preparation obtained as a zymogen in the *Pichia pastoris* expression system followed by autoprocessing to the mature active enzyme [11]. However, the disadvantage of the obtained preparations of cathepsin L was their low stability at acidic pH values (2–3) corresponding to the physiological conditions in the human stomach [12]. 

The aim of this work was to create, based on molecular dynamics simulations and site-specific mutagenesis, recombinant mutant preparations of *T. castaneum* cathepsin L with post-glutamine cleaving activity that are stable in an acidic environment. This peptidase(s) can be further suggested as a lead enzyme for the development of oral medical preparations that fight CD and gluten intolerance in susceptible people.

## 2. Results

Prior to attempting to design a pH-stable version of *Tribolium castaneum* cathepsin L, it would be valuable to uncover the reason for the enzyme’s instability at pH 2. Although molecular dynamics (MD) has proven to be a powerful method for the examination of protein conformational dynamics, it is, unfortunately, unable to explicitly model pH—since the simulated systems’ volumes typically do not exceed much 10 × 10 × 10 nm^3^, there would not be a single free proton (H^+^ or H_3_O^+^) for a whole pH range 2–7. While classical force fields are unable to explicitly model ionization (formation/breaking bonds), a constant-pH MD (CpHMD) approach [13], based on a λ-dynamics formalism [14], is being developed to dynamically assign the charge states of the titratable groups. However, here we used a simpler and widely accepted alternative: the assignment (at the molecular topology level) of the charge state of the ionizable groups, which corresponds to the chosen pH: e.g., at pH 7 acidic residues’ (glutamic and aspartic acids) side chains are negatively charged (deprotonated), while at pH 2 they accept H^+^ and become non-charged. Conversely, histidine is neutral at pH 7 and positively charged (protonated) at pH 2.

**Figure 1:**
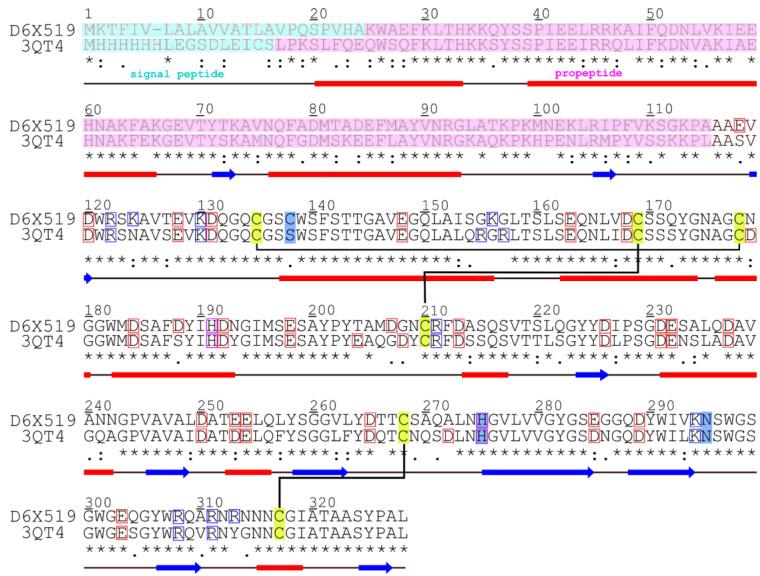
**Sequence alignment for target protein (D6X519) and the template (3QT4).** Secondary structure elements and the sequence conservation line are shown *below* the alignment; disulfide bridges are shown with *black lines* and a *yellow background*. The signal peptide and the propeptide are shaded *cyan* and *pink*, respectively; they are removed from the model. Active site residues are highlighted in *blue*. Asp and Glu residues, which have a charge of −1 at pH 7, are in *red boxes*. Lys and Arg residues have a charge of +1 at both pH values and are in *blue boxes*. His residues have a charge of +1 at pH 2 and are in *purple boxes*. Summation of these charges yields the total protein charge.

**Figure 2:**
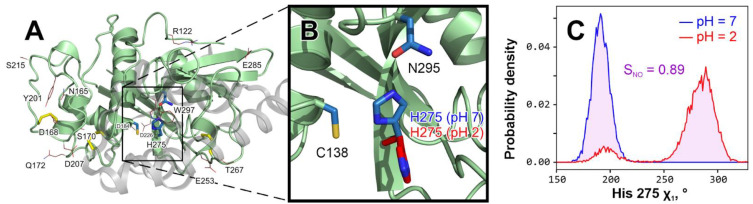
**Catalytic histidine 275 changes conformation at acidic pH.** (**A**). *The overall structure of* Tribolium castaneum *cathepsin L model with residues that “feel” the pH change from 7 to 2 in MD.* The propeptide that keeps an immature enzyme inactive is shown as a semi-transparent gray cartoon and was removed prior to any modeling (Materials and Methods). Disulfide bridges are colored *yellow*. Active site residues (Cys 138, His 275 and Asn 295 [18]) are shown with *blue sticks*. Residues that considerably change χ_1_ distribution at pH 2 compared to pH 7 (*S*_NO_ > 0.5; see panel (**C**)) in at least 2/3 MD replicates are shown with red lines (see Table S1 for a comprehensive list). (**B**). *In-depth analysis reveals that only catalytic His 275 changes may play a role in the enzyme’s activity loss upon acidification.* All other residues from (**A**), except Trp 297, are either small or remote from the active site and solvent-exposed, so their rotation has little effect. In contrast, His 275 keeps a steady in-site position in all pH 7 simulations (blue; see also panel C and Figure 3A, up), while at pH 2 it turns away from the site (red; see also panel C and Figure 3A, down), which may cause activity loss. *C. His 275 χ_1_ torsion angle distribution in indicative pH 7 (*blue*) and pH 2 (*red*) MD trajectories.* To measure the variance between two distributions, we introduce the integral of non-overlapping parameter (*S*_NO_), here equal to 0.89 and represented by pink shading. A full set of such distributions is provided in Figure 3A.

While considering other probable causes of activity loss at pH 2 that are beyond the reach of MD (e.g., protein denaturation and proteolysis), we set a goal of revealing in the MD simulations *conformational* effects that protonated cathepsin L side chains (at pH 2) cause to the enzyme structure considering its mature form without propeptide (Figure 1 and Figure 2A). To achieve this, we modeled cathepsin L at different pH values as follows:

- **pH 7:** all aspartic (14 per protein) and glutamic (10 per protein) acid residue side chains are deprotonated and negatively charged; histidines (2 per protein, including catalytic His 275) are also deprotonated and neutral; lysine (4 per protein) and arginine (5 per protein) are positively charged at pH 7 and 2; protein overall charge is −15.
- **pH “2 (all)”:** all mentioned Asp/Glu/His are protonated; Asp and Glu are neutral and His are positively charged; protein overall charge is +11.
- **pH “2 (his)”:** only catalytic His 275 is protonated; all other residues have their “pH 7” states; protein charge is −14. This “virtual” variant is created to reveal if it’s possible that His 275 pronation itself can cause conformational deviations at acidic pH.

There is one aspect of cysteine proteases that we do not take into account in this research: experiments show that catalytic Cys 138 residue may be deprotonated at neutral pH due to His 275 proximity, which shifts cysteine’s p*K*a by as much as 5, down to 2.5–3.3 for ficin, caricain and papain [15,16], leading to the formation of the ionic pair Cys-S^−^—His-Imidazole^+^. However, the anionic form of cysteine is not implemented in most molecular modeling and dynamics software packages, so proceeding with this variant would lead to separate research focused on the force field parameterization and technical aspects of charged states modeling [17] rather than biochemical work. Due to this reason, in this study, we consider Cys 138 protonated and uncharged.

Ionizable residues and amino acid sequence alignment for TcCathL modeling (detailed in the *Materials and Methods* Section) are highlighted in Figure 1. For all three aforementioned system types, we performed MD calculations for 100 ns, each in three replicates (Table 1, “WT” row, and *Materials and Methods*). Below, we describe the analysis of these calculations and the subsequent work in detail.

### 2.1. Catalytic Histidine 275 Changes Its Conformation at Acidic pH

We performed MD simulations of the cathepsin L molecule at two pH values: 7 and 2, with two types of simulation at the latter pH: pH “2 (all)” and pH “2 (his)” (see text above for explanations of these labels), each type in triplicate, yielding nine trajectories (*Materials and Methods* Section and Table 1 (row “WT”)). The goal was to uncover if there are specific conformational effects to the enzyme’s structure that are caused by protonation of ASP/GLU+HIS residues. We performed a series of analyses that gradually characterized probable effects on the protein structure:

1. Classical root-mean-square deviation (RMSD) analysis (see Figure S1A) revealed that the overall protein structure remains stable at both pH values: we do not see any signs of cathepsin L acid-mediated denaturation. Although, focusing on the active site (residues Cys 138, His 275 and Asn 295 [18]; Figure S1B) reveals a slight instability in most of the pH 2 (both “all” and “his”) trajectories, suggesting that acidification causes not global but local effects in our systems. Furthermore, focusing on His 275 itself (Figure S1C) additionally highlights this effect and reveals a wider RMSD distribution, suggesting multiple rotary transitions.
2. A root-mean-square-fluctuation (RMSF) plot (Figure S2) is designed to uncover protein parts that might become excessively flexible upon acidification. Unexpectedly, almost no residues showed a significant change in RMSF, all being remote from the active site and thus, apparently unable to affect it and reduce the enzyme’s activity.
3. The secondary structure also did not deteriorate much at pH 2, notably, even becoming slightly more stable, although non-significantly (Figure S3).
4. Finally, we focused on the finest parameters of the protein structure, residues’ rotameric states, which are confidently presented by χ_1_ torsion angle distributions. A change in His 275 χ_1_ torsion angle correlates with His 275—Asn 295 distance (not shown) and can serve as a reasonable criterion for determining whether His 275 is located inside the active site or outside it. This analysis revealed that acidification affects the structure of the cathepsin L active site, with the His 275 side chain turning away from the active site in the protonated state (Figure 2B), thus, violating the active site catalytic conformation and reducing the enzyme’s activity.

We characterized each cathepsin residue by the distribution of its χ_1_ torsion angle from each single MD trajectory: either pH 7 (three replicates) or pH “2 all” (another three replicates). For numerical comparison of two distributions, we introduce an integral of non-overlapping (*S*_NO_), which is zero for two identical distributions and unity for two non-overlapping functions (*pink shading* in Figure 2C, illustrating the most unlike His 275 conformations for a pair of pH 7/2 trajectories). Comparing pH 7 and pH “2 all” replicates in pairs, we identified a set of 17 residues with *S*_NO_ > 0.5 in two or three MD replicate pairs (see Figure 2A and Table S1 for a comprehensive list). In-depth analysis revealed that most of these 17 residues are either small or remote from the active site and solvent-exposed, so their rotamer state change upon acidification will have little effect.

**Figure 3:**
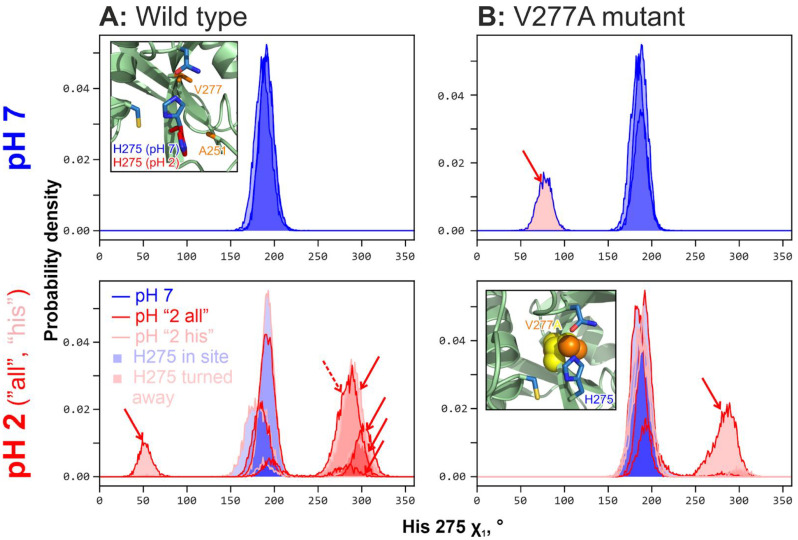
**H275 changes conformation at the acidic pH in cathepsin L wild type but maintains conformation in the V277A mutant.** The four panels show His 275 χ_1_ torsion angle distributions for wild-type (**A**) and V277A mutant (**B**) enzymes at pH 7 (upper panels; blue outline) or pH 2 (both “2 all” (red outline) and “2 his” (pink outline) replicates; lower panels). Blue shading indicates the “native” His 275 rotameric state while red shading indicates its violation in both directions—these are additionally marked with red arrows (broken outline in A; the lower panel corresponds to the red distribution from Figure 2C). Inset in A, upper: cathepsin L active site structure with two His 275 rotamers (analogous to Figure 2A) along with V277 and A251 residues (orange). Inset in B, lower: visualization of V277A mutation, which provides some vacant space inside the active site and thus, may stabilize His 275 in its “native” rotameric state. Note that the V277A mutation dramatically reduces the frequency of rotameric violations for His 275 (one vs. six red arrows).

The only exceptions to the above are Trp 297, located just “above” the active site, and catalytic His 275, which clearly changes its rotameric state in acidic pH (Figure 2). At pH 7, it is stably (in all three replicates) located inside the active site with χ_1_ = 190° ± 9° (see also Figure 3A); conversely, at pH 2 it becomes excessively flexible and often exits the active site (e.g., χ_1_ = 285° ± 12°), which probably leaves the enzyme temporarily dysfunctional (Figure 2B). It should be noted that for ease of calculations, we stacked two χ_1_ diapasons to overcome the jump from +180° to −180°. Of course, this conformational preference is unable to irreversibly damage the enzyme; however, at the same time, it is one of the probable mechanisms of activity loss when pH is reduced. Figure 2C gives an example of a comparison of His 275 χ_1_ distributions at neutral and acidic pH values, revealing *S*_NO_ values as high as 0.89. In some other trajectories, His may be still preferentially located in the active site or be occasionally changing its location (Figure 3A), resulting in lower *S*_NO_ values.

An in-depth comparison of all pH 7 and pH 2 trajectories (Figure 3A) shows that in an acidic environment, His 275 becomes unstable and often leaves the site, probably leading to a loss in activity. In these distributions of χ_1_ torsion angle, *blue shading* indicates native (in-site) His 275 orientation, while a red color pinpoints when it turns away from the site and cannot take part in the catalysis. In all three pH 7 replicates, histidine remains stable (Figure 3A, up), while six pH 2 replicates (3 × “all” and 3 × “his”; Figure 3A, down) reveal a high frequency of conformational violation (additionally shown by *red arrows*). It is worth noting that in the so-called pH “2 his” replicates we see the same effect, suggesting that the protonation of catalytic His 275 itself changes its behavior and forces its side chain to leave the site.

While aware that an increase in His 275 flexibility cannot be the sole cause of the loss of cathepsin L activity upon acidification, nevertheless, we exploit this observation as a working hypothesis in an attempt to undo this malfunction by “fixing” the catalytic His residue in the “right” position by point mutagenesis of the residues in the vicinity of the active site.

### 2.2. His 275 “Fixing” as a Way to Stabilize Cathepsin L

Based upon the aforementioned finding that it is the internal flexibility of His 275 that may be the cause of the loss in cathepsin L activity upon acidification, we decided to design several point mutants that in some sense “fix” this residue inside the active site in the catalytically competent conformation. Close examination of the enzyme structure permitted the identification of position 277 as the most promising position for such an intervention: this residue (valine in wild-type) is next to His 275 in the β-strand (see Figure 3A, *inset*) and certainly influences histidine conformation, while not taking part in the catalysis. The first idea we explored was to put a hydrogen-bonding residue in this position (Asp/Asn/Gln/His/Thr/Tyr) capable of fixing His 275 in the active site by this interaction. The wide range of residue side chain volumes should provide flexibility for the accommodation of such a mutation in the active site vicinity without disturbing the activity. One more possibility explored was A251Y mutation (see Figure 3A, inset), which assumes that the introduction of an aromatic and hydrogen-bonding moiety just after the β-sheet ends may somehow fix His 275 in the site. As a negative control, the V277A variant was added to this list, as according to our initial hypothesis, this mutation should have no effect.

While the preliminary in silico analysis indeed suggested that the V277T and V277D mutations should prevent His 275 from rotating and causing a loss in cathepsin L activity (see Figure S4), in practice, **only the “neutral” V277A mutation proved to be acid resistant in the experimental assessment** (see further sections for biochemical results), exhibiting steady and significant enzymatic activity at pH values as low as 3.

Figure 3, in addition to an in-depth analysis of the wild-type cathepsin MD (panel A), contains a description of the ability of the V277A mutant to resist an acidic environment (panel B). This figure analyzes His 275 rotameric states in neutral (top) and acidic (bottom) conditions. While both versions of the enzyme behave similarly at pH 7 (His 275 exhibits “native” rotameric states, indicated by blue distributions shading; and only minor violation is observed in V277A (red shading and red arrow) [Figure 3, top panels]), at pH 2, a difference emerges: in the wild type, His 275 frequently jumps out of the active site, which probably leads to a loss in activity (red peaks and red arrows in Figure 3A, bottom); conversely, in the V277A mutant, the His 275 side chain remains relatively stable, violating the native state only once (one red arrow in Figure 3B, *bottom* right panel versus six in the bottom left panel).

So why did the so-called “neutral” variant work, while the others did not? Most probably, this is not because of His 275 conformational “fixing” by the hydrogen bond with polar side chains, which may not in fact occur, but because of protein volume reduction in position 277 (V → A), which provides the required room for the His 275 side chain to be accommodated inside the active site at pH 2—a pH at which it gains a charge of +1 and may repel from other charged groups in the vicinity of the active site.

### 2.3. The Probable Role of V277A Substitution in Cathepsin L Stabilization

**Figure 4:**
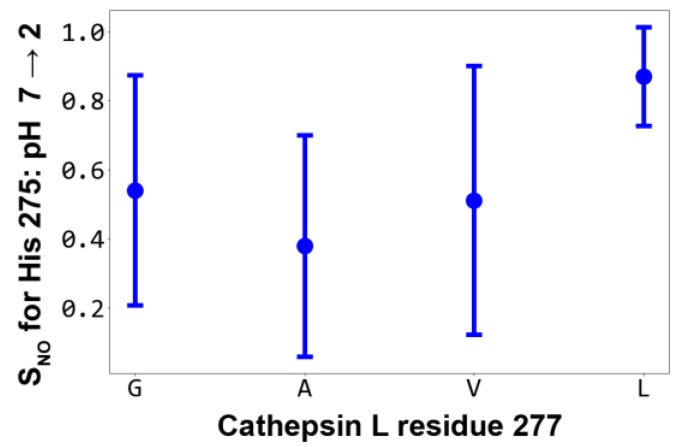
**Alanine in the 277 position of cathepsin L is optimal for His 275 rotameric stabilization (and presumably pH stability).** The graph shows *S*_NO_ for His 275 dependence on the residue 277 identity at the pH switch 7 → 2. Each point is the mean value ± s.d. of three values (*S*_NO_ calculated for 3 × pH 7 and 3 × pH “2 all” replicates). See also Figure S5 for χ_1_ torsion angle distributions.

To demonstrate the significance of the volume of residue 277 for His 275 rotameric stability, we performed additional modeling of V277G (the smallest possible side chain volume) and V277L (a volume increase compared to the WT) mutants at the two pH values and in triplicate (Table 1). Four proteins were compared with a gradually increasing volume of the 277 residue: V277G, V277A, WT (V277) and V277L. The effect of the identity (and volume) of residue 277 on the stabilization of the His 275 rotameric state is summarized by the *S*_NO_ dependence (Figure 4 and Figure S5). Interestingly, the dependence is not monotonic with residue 277 volume: the lowest *S*_NO_ is observed for the V277A variant (0.38 ± 0.32), while both an increase (Val or Leu) and a decrease (Gly) in the side chain volume leads to higher *S*_NO_ values. We conclude that the V277A mutation is optimal for cathepsin L pH stability, which is assessed further experimentally.

### 2.4. Biochemical Evaluation of the Effect of pH on the Activity and Stability of Cathepsin L Mutant Forms

*Tribolium castaneum* cathepsin L variants of the wild type and with the suggested mutations V277T, V277D, V277I, V277A and V277Y were obtained in the form of recombinant proenzymes in the yeast expression system of *Pichia pastoris* (rpTcCathL1 WT, rpTcCathL1 V277T, rpTcCathL1 V277D, rpTcCathL1 V277I, rpTcCathL1 V277A and rpTcCathL1 V277Y). To obtain active enzymes of mutant forms of cathepsin L, autocatalytic processing was carried out according to the method developed for wild-type cathepsin, which resulted in an active enzyme rTcCathL1 WT [11]. However, in practice, we were able to obtain mature forms of recombinant enzymes for only three mutant proteins: rTcCathL1 V277I, rTcCathL1 V277T and rTcCathL1 V277A. Mutant proenzyme rpTcCathL1 V277D was not processed autocatalytically, and no active form was obtained, while rpTcCathL1 V277Y autodegraded instead of processing (that is, the V277D and V277Y mutations interfered with autoprocessing of the cathepsin). Thus, further studies were performed on rTcCathL1 V277I, rTcCathL1 V277T and rTcCathL1 V277A mutant forms in comparison with rTcCathL1 WT.

**Figure 5:**
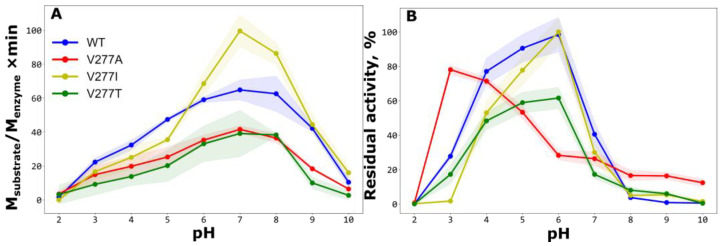
**pH dependence of activity and stability of various cathepsin L forms.** (**A**). Effect of pH on the activities of rTcCathL1 WT, rTcCathL1 V277A, rTcCathL1 V277I and rTcCathL1 V277T with the substrate 0.5 mM Glp–Phe–Gln–pNA in 0.1 M UB, in the presence of 6 mM cysteine and 1 mM EDTA. Note that all enzyme forms have optimum activity at a pH of 7. (**B**). pH stability of the enzymes, which were incubated for 2 h at 37 °C in 0.1 M UB at pH values ranging from 2 to 10 before the pH in all samples was adjusted to 5.6, and the activity was measured as described in (**A**). Relative residual activity values after incubation in a buffer of the appropriate pH were calculated as % of the initial activity at pH 5.6. Note that the highest residual activity after incubation at pH 3 is found with the rTcCathL1 V277A mutant form.

The effect of the pH was studied in two ways: determination of the optimum pH in terms of activity and the pH stability of the enzymes (Figure 5). All mutant proteins, together with the wild-type enzyme, showed maximum activity at neutral pH 7 (Figure 5A), and the highest activity under these conditions was found in mutant enzyme rTcCathL1 V277I. Activity at pH 2 was very low in all forms; however, at pH 3, the activity was already noticeable and amounted to 34% for rTcCathL1 WT and 36% for rTcCathL1 V277A of the maximum activity at pH 7. However, for rTcCathL1 V277A at pH 3, the absolute value of the activity was only one-third of that of rTcCathL1 WT. However, the study of the stability of proteins across two hours of incubation at 37 °C at different pH values showed that rTcCathL1 V277A had the maximum stability at pH 3, at 78% of the initial activity (Figure 5B). The wild-type cathepsin retained only 28% of its activity, the rTcCathL1 V277T mutant form only 17% and the rTcCathL1 V277I form only about 2%. The diagram showing the absolute values of residual activity after incubation of preparations with the same initial activity (Figure S6) shows that despite the lower initial activity at pH 3 (Figure 5A), the mutant form rTcCathL1 V277A after a two-hour incubation at human body temperature has almost twice the activity of rTcCathL1 WT. In the human stomach, pH is normally between 1.5 and 3.5, and food digestion lasts 1–4 h [12]; therefore, pH 3 is quite suitable for effective gliadin hydrolysis by rTcCathL1 V277A of at least 2 h. We suggest this protein as a leading candidate glutenase for the development of oral medical preparations that fight CD and gluten intolerance in susceptible people (patent application No. 2023115446 “Obtaining Recombinant Acid-Stable Cathepsin L (Mutant Form) and Methods of Its Application”, Federal Service for Intellectual Property, Russia). 

It should be noted that maximum pH stability for all other forms was observed at pH 6, which is consistent with the physiological conditions for the functioning of digestive cathepsin L in the *T. castaneum* midgut at pH 5.6. Long-term incubation for 2 h at pH 6 did not affect the activity of rTcCathL1 WT and rTcCathL1 V277I, since the residual activity under these conditions was close to 100%. Only in rTcCathL1 V277T did the activity drop to 62% (Figure 5A).

## 3. Discussion

One of the main reasons for the decrease in the activity of peptidases in an acidic environment is structural changes in the enzyme. We employed MD simulations to uncover probable conformational violations that accompany acidification and revealed that the proposed computational protocol is quite reliable for this purpose. Thus, at the timescale of the modeling, there were no signs of the enzyme unfolding. The main result we discovered was that catalytic His 275—the core amino acid residue of the active site of the cysteine proteinases—constantly left the active site in acidic conditions, thereby apparently making catalysis impossible. The only way that classical MD can account for changes due to pH is by varying the charge state of the ionizable residues—which was implemented in our “pH 7” and “pH 2” trajectories. From Figure 3A, one can note that at pH 2, the protonated His 275 is unstable and often leaves the active site (*red distributions*). It is important to note that even in the “pH 2 (his)” trajectories, when the only protonated residue in the structure is His 275, the effect persists, probably indicating repulsion of the histidine side chain (which gained a positive charge at this pH) from the vicinity of the active site. While aware that His 275 flexibility increase cannot be the sole cause of cathepsin L activity loss upon acidification, nevertheless, we suggest that the conformation of its side chain may be used as a signature of enzyme instability at low pH values.

Given this, we tried to engineer point amino acid substitutions in the vicinity of the active site that might stabilize the His 275 conformation. The most suitable substituted residue for this intervention was found to be the Val 277 (Figure 3B, inset). Unexpectedly, the most promising candidates (e.g., V277T (Figure S4), which was suggested to form an H-bond with His 275 and “hold” it in place) did not work (Figure 5B). Conversely, the proposed mutation V277A, which is “neutral” in terms of its effect on the H-bonding pattern of His 275, leads to a freeing up of space at the site that can also affect the behavior of the catalytic residue. Subsequent experimental testing revealed that the mutant V277A became pH stable while still possessing reasonable activity after exposure to the acidic milieu (Figs. 3B and 5B). Compared to two other mutations tested in silico, V277G (with the shortest possible side chain) and V277L (with increased volume relative to the wild type), the V277A mutant displayed the lowest *S*_NO_ parameter for His 275 (Figure 4), suggesting that this mutant had the most stable His 275 conformation. One probable cause for this is simply the volume of the 277 residue, which is optimal in the case of V277A for providing the needed room for the accommodation of His 275 in the active site in both neutral and acidic environments.

The unexpected results obtained in this study show that the rational design of the enzymes still represents an area of uncertainty and requires many trial-and-error cycles incorporating both design attempts and experimental validation. Anyhow, the in silico design employed here made it possible to quickly evaluate different scenarios of rearrangements at the enzyme active site at different pH values. As a result, only a very limited number of residues—candidates for point mutagenesis and experimental testing of activity—were proposed. Most significantly, one of these variants—V277A—displayed the desired increase in enzyme stability at low pH. This is important from both the fundamental and practical points of view. In the case of the former, this provides an additional approach to solving a very complex problem in the computational design of enzymes with modified pH sensitivity. In the case of the latter, this leads to a real protein that can be further employed to combat CD and gluten intolerance in susceptible people.

## 4. Materials and Methods

### 4.1. Systems Setup, Molecular Dynamics and Data Analysis

To assess the effect of a change in pH from 7 to 2 on cathepsin L conformational dynamics and active site structure, we set up MD calculations at different pH values, which was achieved by the manual assignment of the charge states of titratable residues (ASP + GLU and HIS). To gain enough statistics, calculations were performed in triplicate with similar but individual starting states: (1) a homology model built in this work; (2) a structure predicted with the AlphaFold tool [19]; and (3) a model downloaded from the SwissModel database [20]. All three models are very similar: RMSD values for models pairs 1–2, 2–3 and 1–3, calculated for the backbone/all heavy atoms, are 0.69/3.06 Å, 0.64/2.03 Å and 0.22/2.96 Å, respectively. *Tribolium castaneum* cathepsin L target sequence was taken from Uniprot (Id: D6X519, NCBI Id: NP_001164001); the template for homology modeling was found using BLAST. This is a procathepsin L mutant from the *Tenebrio molitor* larval midgut with catalytic cysteine replaced with serine to stabilize the protein (PDB entry 3QT4) [21]. Homology modeling was performed with MODELLER [22] using the alignment shown in Figure 1. *N*-terminal signal peptide and the propeptide with a total length of 115 residues were removed from the model (Figure 1 and Figure 2A), as after folding, this fragment is autocatalytically cleaved, yielding an active mature form of the enzyme. Point mutants were constructed by the Pymol [23] *mutagenesis* option. Systems with only one MD replicate calculated (see Table 1) were based on the “Modeller” starting structure.

MD simulations were performed using the GROMACS software package version 2020.6 [24] and CHARMM36 [25] (WT and V277A “Modeller” replicates) or AMBER99SB-ILDN [26] (for all other replicates and mutants) force field parameters (different force fields were selected to bring variety to the dataset; in practice, they produced very similar results). The complete list of modeled systems, with an aggregate MD time of 10 µs, is provided in Table 1. An integration time step of 2 fs was used, and 3D periodic boundary conditions were imposed. An explicit solvent model (SPC/E) [27] and Na^+^ and Cl^−^ ion parameters for counter ions were used [28]. Simulations were performed at a temperature of 300 K maintained using the V-rescale algorithm [29]. A pressure of 1 bar was maintained using the Parrinello–Rahman (wild type, V277A, V277G, V277L) [30] and Berendsen (other mutants) [31] algorithms.

The analysis of RMSD, RMSF and secondary structure was performed using the *gmx rms*, *gmx rmsf* and *gmx dssp* utilities, respectively. Dihedral angle data were obtained using *gmx chi*; to overcome the jump between +180° and −180°, angle values were normalized to a range of 0–360° by doubling and stacking the data. The integral of non-overlapping (*S*_NO_) parameter for two χ_1_-angle distributions is defined as the difference between the two areas under the graphs divided by the respective sum. Molecular graphics were rendered using PyMOL v. 2.6 [23].

### 4.2. Production of Recombinant Mutant Forms of Procathepsin L and Their Processing

The cloning, expression, and isolation of the wild-type *T. castaneum* procathepsin L (TcCathL1, Uniprot D6X519, NCBI NP_001164001) were performed as described in a previous study [11]. The pTcCathL1 WT gene was cloned into the pPICZalphaA vector, expressed in *P. pastoris* GS115-II-3 transformants, and recombinant procathepsin L (rpTcCathL1 WT) was isolated from the culture medium by ammonium sulfate precipitation.

Site-directed mutagenesis of the prTcCathL1 WT gene and the production of pPICZalfaA vectors containing mutant genes were carried out according to standard methods [32]. The expression and isolation of recombinant mutant forms of pTcCathL1—rpTcCathL1 WT, rpTcCathL1 V277T, rpTcCathL1 V277D, rpTcCathL1 V277I, rpTcCathL1 V277A and rpTcCathL1 V277Y—was also carried out in the *P. pastoris* expression system, similarly to the production of prTcCathL1 WT.

Processing of the wild-type and mutant forms of procathepsin L was performed autocatalytically by incubation at pH 4.0 and 37 °C for 60–150 min as described in [11].

### 4.3. Assay of Peptidase Activity

The enzymatic activity was measured in a 96-well plate at 405 nm according to [10] in the presence of 6 mM cysteine and 1 mM EDTA in 0.1 M acetate–phosphate–borate universal buffer (UB) [33] at pH 5.6 (the physiological pH of *T. castaneum* midgut [34]) using a selective chromogenic substrate 0.5 mM Glp–Phe–Gln–pNA [35]. The reaction mixture was incubated at 37 °C. The activity was calculated in nmol/min from the initial rate of hydrolysis after titration of peptidase active sites with the irreversible inhibitor E-64 according to [36].

### 4.4. Assay of the Effect of pH on the Activity and Stability of Cathepsin L

The effect of pH on cathepsin L activity was studied in 0.1 M UB with pH values from 2 to 10 in the presence of 6 mM cysteine and 1 mM EDTA. Activity was measured with the 0.5 mM Glp–Phe–Gln–pNA substrate, as described above, and calculated as specific activity.

The pH stability of the enzyme was tested after 2 h incubation at 37 °C in 0.1 M UB at pH values ranging from 2 to 10. Activity was measured twice: before incubation at different pH and after incubation. In both cases, measurements were performed at a physiological pH of 5.6, as described above. Stability was evaluated by relative residual activity before and after incubation and expressed as %%, or by the level of absolute residual activity after incubation.

All calculations of comparative measurements were performed by equalizing the concentrations of all enzymes according to the results of active site titration measurements (as described above).

## 5. Conclusions

In this work, we made an advance in a quest for oral enzyme therapeutics that may be administered to people susceptible to CD and NCGS to allow them to digest immunogenic prolamin peptides. We proposed that digestive cathepsin L from the insect pest *Tribolium castaneum*, which has high post-glutamine cleaving activity, may serve as a basis for this quest, and computer modeling of the enzyme dynamics may provide a clue for mutagenesis conferring pH stability on the engineered protein. From computational simulations, we discovered that, at first glance, insignificant change—the V277A mutation—somehow stabilizes the active site of the enzyme during a change in pH from 7 to 2, probably by providing the needed room for the catalytic His 275 residue to remain within the active site. A subsequent biochemical evaluation revealed that, although losing some activity, cathepsin L V277A indeed shows increased stability at the acidic pH 3 compared to the wild type, and thus, is a leading candidate enzyme for use in the treatment of CD.

## 6. Patents

An application for an invention No. 2023115446 “Obtaining Recombinant Acid-Stable Cathepsin L (Mutant Form) and Methods of Its Application” has been submitted to the Federal Service for Intellectual Property, Russia.

## Figures and Tables

**Table 1:** **MD simulations of cathepsin L variants in water conducted in this work.** To explore pH effects on enzyme dynamics and their probable recovery by point mutations, cathepsin L wild type (WT) and several mutant variants were modeled at different pH values (2 or 7) by assigning the charge states of Asp, Glu and His residues. pH index “2 (his)” means that only catalytic His 275 was protonated, leaving all other groups in the “pH 7” state. Where three replicates are specified, starting coordinates were assigned from different cathepsin L models (see *Materials and Methods* text). See Figure 1 for a full list of charge-forming residues.

Type	рН	# of Replicates	Charge	MD Length, ns
WT	7	3	−15	100
	2 (all)	3	+11	100
	2 (his)	3	−14	100
V277A	7	3	−15	100
	2 (all)	3	+11	100
	2 (his)	3	−14	100
V277G	7	3	−15	100
	2 (all)	3	+11	100
V277L	7	3	−15	100
	2 (all)	3	+11	100
V277D	7	1	−16	500
	2 (all)	1	+11	500
V277H	7	1	−15	500
	2 (all)	1	+12	500
V277N	7	1	−15	500
	2 (all)	1	+11	500
V277Q	7	1	−15	500
	2 (all)	1	+11	500
V277T	7	1	−15	500
	2 (all)	1	+11	500
V277Y	7	1	−15	500
	2 (all)	1	+11	500
A251Y	7	1	−15	500
	2 (all)	1	+11	500

## Data Availability

The preview of the MD data is available at Zenodo: https://doi.org/10.5281/zenodo.8199670.

## Supplementary Materials

The following supporting information can be downloaded at: https://www.mdpi.com/article/10.3390/ijms241512369/s1.

## References

[1] Catassi C., Verdu E.F., Bai J.C., Lionetti E. (2022). Coeliac Disease. Lancet.

[2] Lebwohl B., Ludvigsson J.F., Green P.H.R. (2015). Celiac Disease and Non-Celiac Gluten Sensitivity. BMJ.

[3] Barbaro M.R., Cremon C., Stanghellini V., Barbara G. (2018). Recent Advances in Understanding Non-Celiac Gluten Sensitivity. F1000Research.

[4] Dunaevsky Y.E., Tereshchenkova V.F., Belozersky M.A., Filippova I.Y., Oppert B., Elpidina E.N. (2021). Effective Degradation of Gluten and Its Fragments by Gluten-Specific Peptidases: A Review on Application for the Treatment of Patients with Gluten Sensitivity. Pharmaceutics.

[5] Wei G., Helmerhorst E.J., Darwish G., Blumenkranz G., Schuppan D. (2020). Gluten Degrading Enzymes for Treatment of Celiac Disease. Nutrients.

[6] Darwish G., Helmerhorst E.J., Schuppan D., Oppenheim F.G., Wei G. (2019). Pharmaceutically Modified Subtilisins Withstand Acidic Conditions and Effectively Degrade Gluten in Vivo. Sci. Rep..

[7] Gordon S.R., Stanley E.J., Wolf S., Toland A., Wu S.J., Hadidi D., Mills J.H., Baker D., Pultz I.S., Siegel J.B. (2012). Computational Design of an α-Gliadin Peptidase. J. Am. Chem. Soc..

[8] Wolf C., Siegel J.B., Tinberg C., Camarca A., Gianfrani C., Paski S., Guan R., Montelione G., Baker D., Pultz I.S. (2015). Engineering of Kuma030: A Gliadin Peptidase That Rapidly Degrades Immunogenic Gliadin Peptides in Gastric Conditions. J. Am. Chem. Soc..

[9] CTG Labs—NCBI. https://www.clinicaltrials.gov/study/NCT03701555.

[10] Dvoryakova E.A., Vinokurov K.S., Tereshchenkova V.F., Dunaevsky Y.E., Belozersky M.A., Oppert B., Filippova I.Y., Elpidina E.N. (2022). Primary Digestive Cathepsins L of Tribolium castaneum Larvae: Proteomic Identification, Properties, Comparison with Human Lysosomal Cathepsin L. Insect Biochem. Mol. Biol..

[11] Dvoryakova E.A., Klimova M.A., Simonyan T.R., Dombrovsky I.A., Serebryakova M.V., Tereshchenkova V.F., Dunaevsky Y.E., Belozersky M.A., Filippova I.Y., Elpidina E.N. (2022). Recombinant Cathepsin L of Tribolium castaneum and Its Potential in the Hydrolysis of Immunogenic Gliadin Peptides. Int. J. Mol. Sci..

[12] Helmenstine A.M. What Is the pH of the Stomach?. https://www.thoughtco.com/ph-of-the-stomach-608195.

[13] Aho N., Buslaev P., Jansen A., Bauer P., Groenhof G., Hess B. (2022). Scalable constant pH molecular dynamics in GROMACS. J. Chem. Theory Comp..

[14] Kong X., Brooks III C.L. (1996). λ-dynamics: A new approach to free energy calculations. J. Chem. Phys..

[15] Pinitglang S., Watts A.B., Patel M., Reid J.D., Noble M.A., Gul S., Bokth A., Naeem A., Patel H., Thomas E.W. (1997). A classical enzyme active center motif lacks catalytic competence until modulated electrostatically. Biochemistry.

[16] Harris T.K., Turner G.J. (2002). Structural basis of perturbed pKa values of catalytic groups in enzyme active sites. IUBMB life.

[17] Awoonor-Williams E., Golosov A.A., Hornak V. (2023). Benchmarking In Silico Tools for Cysteine pKa Prediction. J. Chem. Inf. Model..

[18] Rawlings N.D., Barrett A.J. (2013). Introduction: The Clans and Families of Cysteine Peptidases. Handbook of Proteolytic Enzymes.

[19] Jumper J., Evans R., Pritzel A., Green T., Figurnov M., Ronneberger O., Tunyasuvunakool K., Bates R., Žídek A., Potapenko A. (2021). Highly Accurate Protein Structure Prediction with AlphaFold. Nature.

[20] Waterhouse A., Bertoni M., Bienert S., Studer G., Tauriello G., Gumienny R., Heer F.T., de Beer T.A.P., Rempfer C., Bordoli L. (2018). SWISS-MODEL: Homology Modelling of Protein Structures and Complexes. Nucleic Acids Res..

[21] Beton D., Guzzo C.R., Ribeiro A.F., Farah C.S., Terra W.R. (2012). The 3D Structure and Function of Digestive Cathepsin L-like Proteinases of Tenebrio molitor Larval Midgut. Insect Biochem. Mol. Biol..

[22] Fiser A., Sali A. (2003). Modeller: Generation and Refinement of Homology-Based Protein Structure Models. Methods Enzymol..

[23] (2023). The PyMOL Molecular Graphics System.

[24] Abraham M.J., Murtola T., Schulz R., Páll S., Smith J.C., Hess B., Lindahl E. (2015). GROMACS: High Performance Molecular Simulations through Multi-Level Parallelism from Laptops to Supercomputers. SoftwareX.

[25] Huang J., MacKerell A.D. (2013). CHARMM36 All-Atom Additive Protein Force Field: Validation Based on Comparison to NMR Data. J. Comput. Chem..

[26] Lindorff-Larsen K., Piana S., Palmo K., Maragakis P., Klepeis J.L., Dror R.O., Shaw D.E. (2010). Improved Side-Chain Torsion Potentials for the Amber ff99SB Protein Force Field. Proteins.

[27] Berendsen H.J.C., Grigera J.R., Straatsma T.P. (1987). The Missing Term in Effective Pair Potentials. J. Phys. Chem..

[28] Beglov D., Roux B. (1994). Finite Representation of an Infinite Bulk System: Solvent Boundary Potential for Computer Simulations. J. Chem. Phys..

[29] Bussi G., Donadio D., Parrinello M. (2007). Canonical Sampling through Velocity Rescaling. J. Chem. Phys..

[30] Parrinello M., Rahman A. (1981). Polymorphic Transitions in Single Crystals: A New Molecular Dynamics Method. J. Appl. Phys..

[31] Berendsen H.J.C., Postma J.P.M., Van Gunsteren W.F., DiNola A., Haak J.R. (1984). Molecular dynamics with coupling to an external bath. J. Chem. Phys..

[32] (2015). QuikChange II XL Site-Directed Mutagenesis Kit; Catalog #200521 and #200522.

[33] Frugoni J.A.C. (1957). Tampone Universale Di Britton E Robinson a Forza Ionica Costante. Gazz. Chim. Ital..

[34] Vinokurov K.S., Elpidina E.N., Oppert B., Prabhakar S., Zhuzhikov D.P., Dunaevsky Y.E., Belozersky M.A. (2006). Fractionation of Digestive Proteinases from Tenebrio molitor (Coleoptera: Tenebrionidae) Larvae and Role in Protein Digestion. Comp. Biochem. Physiol. B Biochem. Mol. Biol..

[35] Filippova I.Y., Dvoryakova E.A., Sokolenko N.I., Simonyan T.R., Tereshchenkova V.F., Zhiganov N.I., Dunaevsky Y.E., Belozersky M.A., Oppert B., Elpidina E.N. (2020). New Glutamine-Containing Substrates for the Assay of Cysteine Peptidases from the C1 Papain Family. Front Mol Biosci.

[36] Semashko T.A., Vorotnikova E.A., Sharikova V.F., Vinokurov K.S., Smirnova Y.A., Dunaevsky Y.E., Belozersky M.A., Oppert B., Elpidina E.N., Filippova I.Y. (2014). Selective Chromogenic and Fluorogenic Peptide Substrates for the Assay of Cysteine Peptidases in Complex Mixtures. Anal. Biochem..

