# Supplementary material for: Fighting Celiac Disease: Improvement of pH Stability of Cathepsin L In Vitro by Computational Design
Source: Int J Mol Sci. 2023 Aug 2;24(15):12369. doi: 10.3390/ijms241512369 (PMC10418366; doi:10.3390/ijms241512369)
Supplement: Supplementary file 1 [file ijms-24-12369-s001.zip › ijms-2503699-supplementary.pdf]

Fighting Celiac Disease: Improvement of pH Stability of Cathepsin L *In Vitro* by Computational Design

Anton O. Chugunov [1,2,*], Elena A. Dvoryakova [3], Maria A. Dyuzheva [1,4], Tatyana R. Simonyan [5], Valeria F. Tereshchenkova [5], Irina Yu. Filippova [5], Roman G. Efremov [1,2,6] and Elena N. Elpidina [3]

[1] M.M. Shemyakin and Yu. A. Ovchinnikov Institute of Bioorganic Chemistry, Russian Academy of Sciences, 117997 Moscow, Russia
[2] Moscow Institute of Physics and Technology (State University), 141701 Dolgoprudny, Russia
[3] A.N. Belozersky Institute of Physico-Chemical Biology, M.V. Lomonosov Moscow State University, 119991 Moscow, Russia
[4] Higher Chemical College of the Russian Academy of Sciences, D. Mendeleev University of Chemical Technology, 125047 Moscow, Russia
[5] Department of Chemistry, M.V. Lomonosov Moscow State University, 119991 Moscow, Russia
[6] Department of Applied Mathematics, National Research University Higher School of Economics, 101000 Moscow, Russia
* Correspondence: batch2k@yandex.ru

Supplementary information

Table S1. The residues' sensitivity to pH changing. There are 17 residues with S_{NO} > 0.5 (*italicized* in the third column) in two or three replicas pairs pH 7 / pH "2 (all)". The residues' χ_1-angle values are presented as mean ± s.d. for three joint replicas (pH 7) or for each replica (pH 2 "all"). Only the dominant peak was selected, or two in case there were two similar. Some distributions (Asn 168, Asp 168) have very close, but narrow peaks, which determines the high value of S_{NO}.

Residue	Frequency	S_{NO}	Angle at pH 7	Angle at pH 2 "all"
Ser 170	3	*0.52*<br>*0.76*<br>*0.98*	71°±11°	294°±16°, 69°±8°<br>305°±9°<br>193°±10°
Asp 226	3	*0.95*<br>*0.84*<br>*0.97*	56°±11°	289°±10°<br>186°±13°<br>293°± 9°
Arg 122	3	*0.93*<br>*0.53*<br>*0.65*	291°±8°	182°±10°<br>186°±10°,283°±9°<br>58°±11°
Asp 184	3	*0.91*<br>*0.93*<br>*0.63*	53°±11°	286°±10°<br>291°±9°<br>297°±8°
Asp 289	2	*0.94*<br>*0.92*<br>0.06	307°±11°	184°±9°<br>183°±10°<br>293°±9°
Ser 215	2	*0.53*<br>*0.55*<br>0.43	70°±9°	304°±12°<br>304°±12°<br>56°±9°
Ser 233	2	*0.61*<br>*0.59*<br>0.47	74°±11°	301°±13°<br>302°±12°<br>55°±10°
Gln 172	2	*0.70*<br>0.44<br>*0.58*	298°±11°	72°±11°<br>188°±11°<br>67°±9°
Glu 253	2	*0.87*<br>*0.53*<br>0.27	294°±10°	78°±8°<br>188°±11°<br>294°±10°
Asp 207	2	*0.91*<br>*0.95*<br>0.44	52°±10°	193°±15°<br>192°±13°<br>298°±9°
Thr 267	2	*0.51*<br>0.43<br>*0.57*	62°±13°	56°±9°<br>57°±9°<br>48°±11°
His 275	2	*0.89*<br>0.11<br>*0.54*	190°±9°	285°±12°<br>192°±9°<br>185°±9°, 303°±10°
Glu 285	2	*0.64*<br>*0.56*<br>0.49	184°±11°	288°±10°<br>286°±11°<br>292°±10°, 181°±12°
Tyr 201	2	*0.57*<br>*0.65*<br>0.25	204°±15°	191°±8°<br>285°±12°, 195°±10°<br>194°±9°
Trp 297	2	*0.61*<br>0.28<br>*1.00*	302°±9°	203°±17°, 298°±12°<br>297°±9°<br>50°±10°
Asn 165	2	*0.54*<br>*0.65*<br>0.17	189°±10°	205°±8°<br>176°±10°<br>185°±9°
Asp 168	2	*0.55*<br>*0.55*<br>0.49	298°±10°	290°±10°<br>292°±7°<br>294°±8°

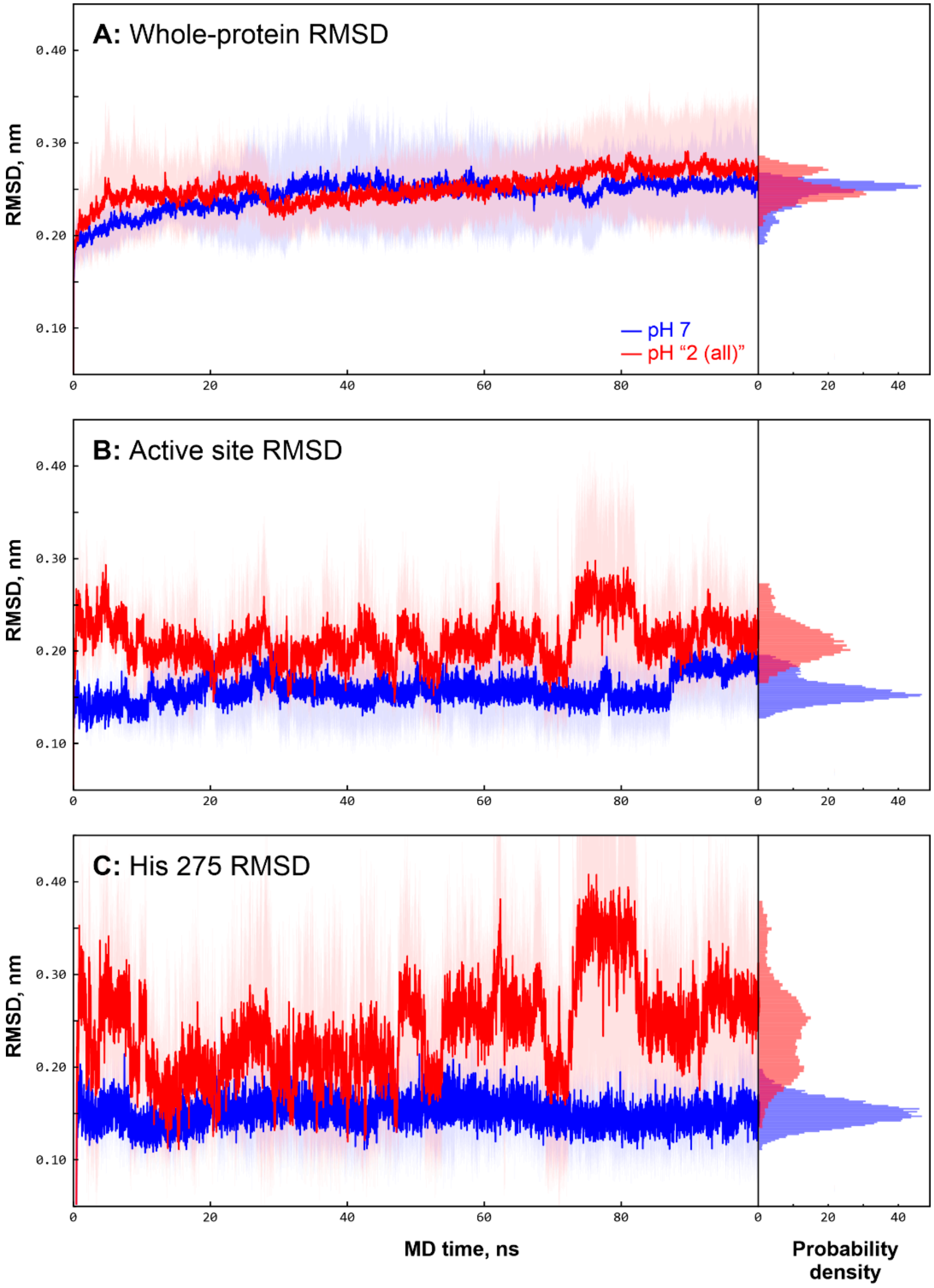

Figure S1. Cathepsin L Root Mean Square Deviation (RMSD). A. Whole protein RMSD time series at pH 7 (*blue*) and pH 2 (*red*), averaged over three trajectories each with standard deviation shown by *shading*. *To the right:* RMSD distribution over trajectories. No significant difference between two pH values is observed. B. Active site only RMSD after least-square fit over protein backbone atoms. Note that pH 2 values are slightly larger, indicating greater active site distortion. C. His 275 only RMSD after least-square fit over protein backbone atoms. Note larger values and wider distribution for pH 2 trajectories.

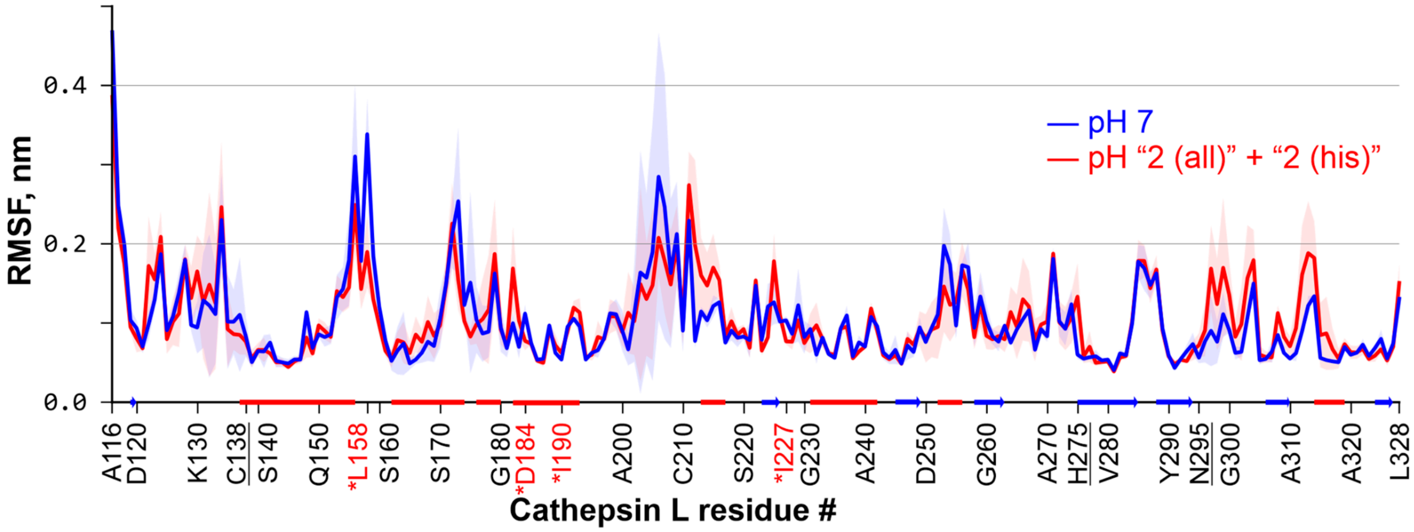

Figure S2. Cathepsin L Root Mean Square Fluctuation (RMSF) at pH 7 and pH 2. RMSF was calculated for three pH 7 (*blue*) and six pH 2 (3 × "all" and 3 × "his"; *red*) MD trajectories; mean values are reported by *plots* and standard deviations — by *shadings*. Secondary structure elements are shown *below. Asterisk* and *red color* in residues' labels mark those with significant ($p < 0.05$ in t-test) RMSF difference between the two pH values. Note that there are just a few of them, quite remote from the active site (*underlined*) and apparently unable to affect enzyme activity.

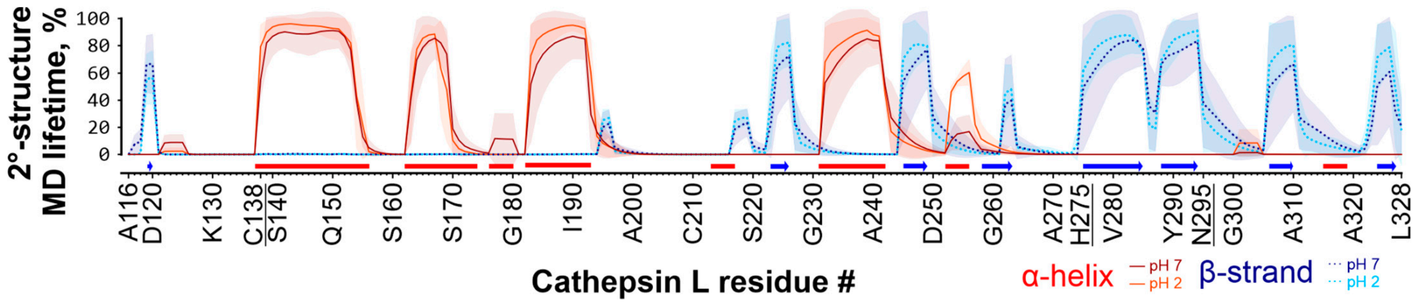

Figure S3. Cathepsin L secondary (2°) structure is calculated over three MD replicas for each pH 7 and pH "2 (all)" value and plotted as mean MD lifetime and standard deviations (*shadings*) of two major elements: α-helices (*red solid lines*) and β-strands (*blue dotted lines*). 2°-structure in the initial model (identical to Figs. 1 and S2) is shown *below*. Note a slightly more stable (although, apparently non-significantly) structure at pH 2.

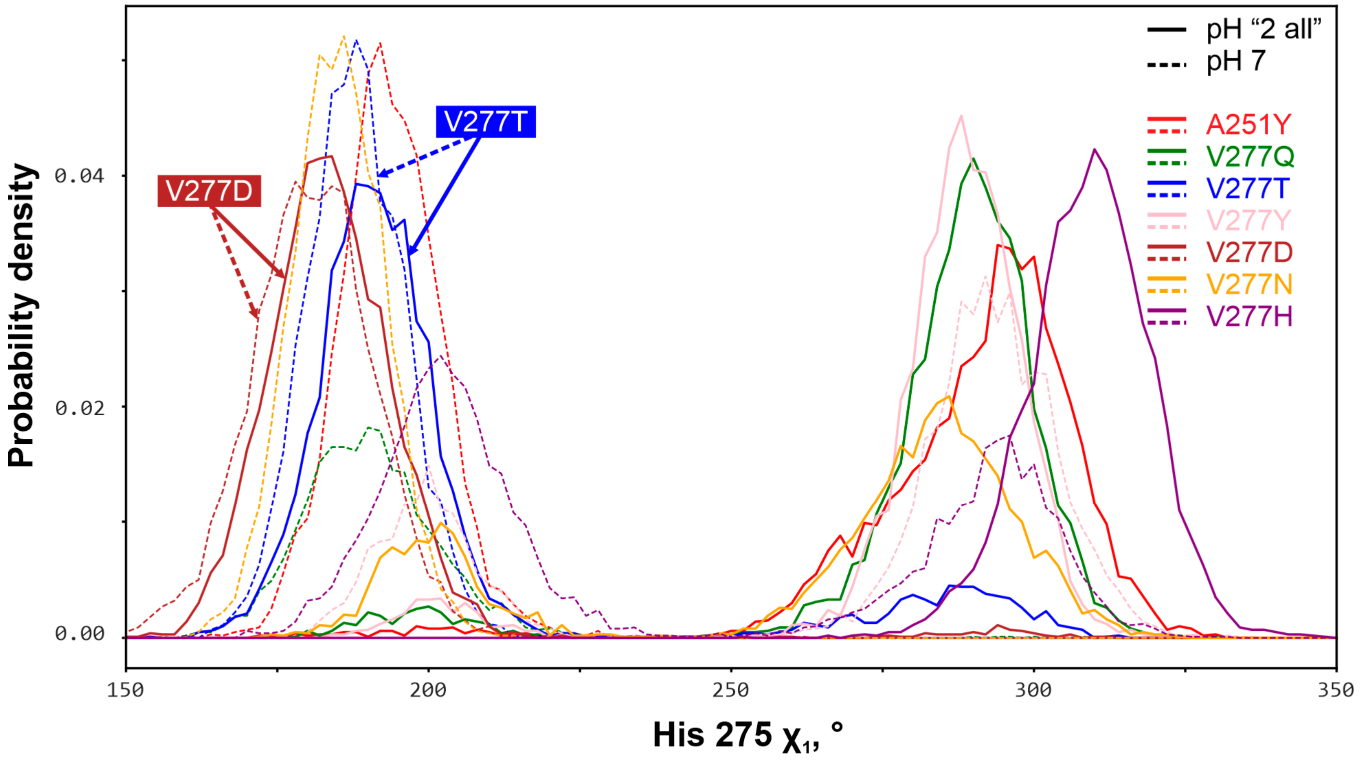

Figure S4. Preliminary *in silico* assessment of cathepsin L mutants to stabilize His 275 rotameric state upon acidification. Graphs represent His 275 χ_1 torsion angle distributions for seven cathepsin L mutants: six in the V277 position (to Q, T, Y, D, N, H) and one A251Y; each modeled at two pH values. We suggested that mutation has stabilizing effect when both pH distributions are alike (and S_{NO} are close to zero), as for V277D and V277T (additionally *labeled*; S_{NO} are as little as 0.12 and 0.22, respectively); since in wild type His 275 changes rotameric state during pH 7 → 2 switch (S_{NO}= 0.89; see Fig. 2B, C in the main text). (Other S_{NO} values in increasing order are: 0.31 for V277Y; 0.73 for V277H; 0.88 for V277N; 0.95 for V277Q; and 0.98 for A251Y.) However, experimental testing revealed that none of these actually have acid resistance, apart from V277A mutant that was initially selected just as "negative control", but indeed had pH-stabilizing effect on cathepsin L (see main text).

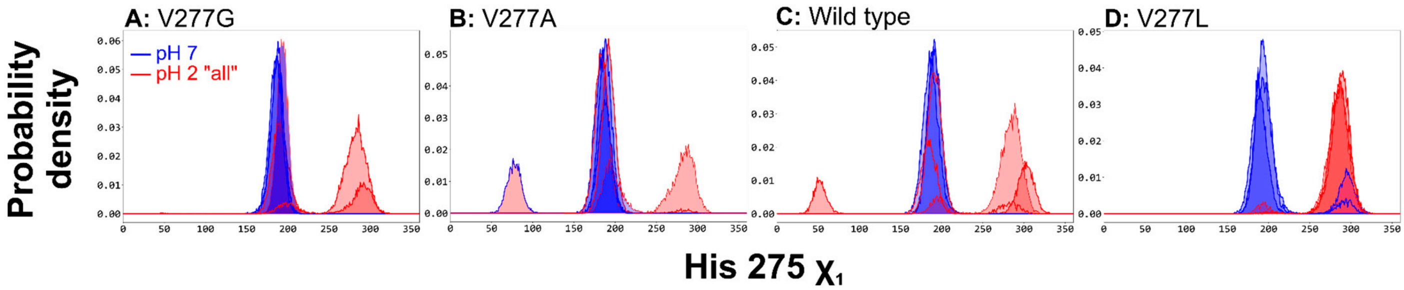

Figure S5. His 275 χ_1 torsion angle distributions for four cathepsin versions: V277G mutant (A), V277A mutant (B), wild type (V277; C), and V277L mutant (D) at pH 7 (*blue outline*) and pH 2 (*red outline*). *Blue shading* points out "native" His 275 rotameric state; *red shading* — its violation to both directions. S_{NO} values are: A — 0.36, 0.34, 0.93; B — 0.05, 0.69, 0.40; C — 0.54, 0.89, 0.11; D — 0.99, 0.71, 0.91. S_{NO} summary is provided in Fig. 4 in the main text.

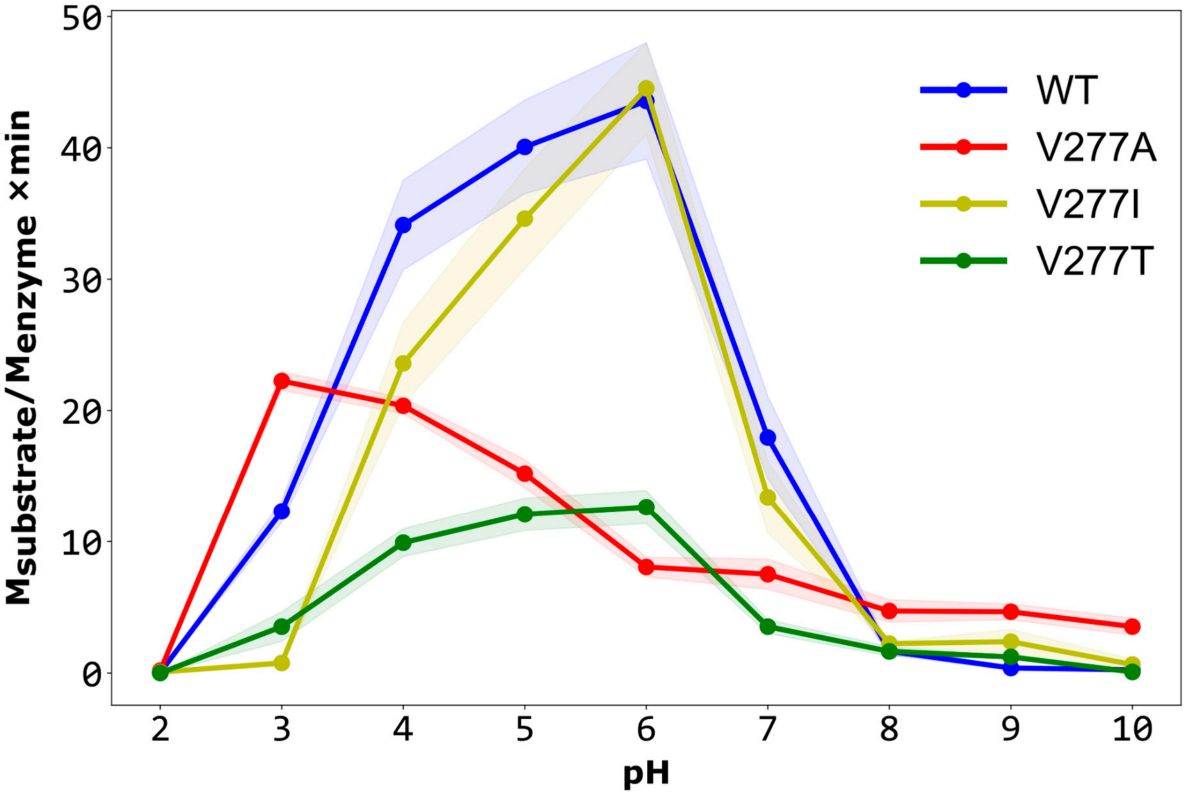

Figure S6. pH-Stability of various cathepsin L forms. pH-stability was measured for the enzymes rTcCathL1 WT, rTcCathL1 V277A, rTcCathL1 V277I, and rTcCathL1 V277T that were incubated for 2 h in 0.1 M UB, with pH values ranging from 2 to 10 at 37 ℃. Then the pH in all samples was adjusted to 5.6, and the activity was measured with the substrate 0.5 mM Glp–Phe–Gln–pNA in 0.1 M UB, in the presence of 6 mM cysteine and 1 mM EDTA. Note that the maximal residual activity after incubation at pH 3 was found for the rTcCathL1 V277A mutant form.
